# A Systematic Comparison of Depth Map Representations for Face Recognition

**DOI:** 10.3390/s21030944

**Published:** 2021-01-31

**Authors:** Stefano Pini, Guido Borghi, Roberto Vezzani, Davide Maltoni, Rita Cucchiara

**Affiliations:** 1 DIEF—Dipartimento di Ingegneria Enzo Ferrari, Università Degli Studi di Modena e Reggio Emilia, 41125 Modena, Italy; s.pini@unimore.it (S.P.); roberto.vezzani@unimore.it (R.V.); rita.cucchiara@unimore.it (R.C.); 2 DISI—Dipartimento di Informatica-Scienza e Ingegneria, Università di Bologna, 47521 Cesena, Italy; davide.maltoni@unibo.it; 3 AIRI—Artificial Intelligence Research and Innovation Center, Università Degli Studi di Modena e Reggio Emilia, 41125 Modena, Italy

**Keywords:** face recognition, depth maps, depth sensors, depth map representations, surface normal, point cloud, voxel, dataset

## Abstract

Nowadays, we are witnessing the wide diffusion of active depth sensors. However, the generalization capabilities and performance of the deep face recognition approaches that are based on depth data are hindered by the different sensor technologies and the currently available depth-based datasets, which are limited in size and acquired through the same device. In this paper, we present an analysis on the use of depth maps, as obtained by active depth sensors and deep neural architectures for the face recognition task. We compare different depth data representations (depth and normal images, voxels, point clouds), deep models (two-dimensional and three-dimensional Convolutional Neural Networks, PointNet-based networks), and pre-processing and normalization techniques in order to determine the configuration that maximizes the recognition accuracy and is capable of generalizing better on unseen data and novel acquisition settings. Extensive intra- and cross-dataset experiments, which were performed on four public databases, suggest that representations and methods that are based on normal images and point clouds perform and generalize better than other 2D and 3D alternatives. Moreover, we propose a novel challenging dataset, namely MultiSFace, in order to specifically analyze the influence of the depth map quality and the acquisition distance on the face recognition accuracy.

## 1. Introduction

In the computer vision field, Face Recognition is a widely studied task and impressive results have been obtained in the RGB domain [1,2,3], specially with frontal face poses and good lighting conditions. Moreover, a substantial improvement has been introduced by the adoption of (very) deep neural networks [4,5,6] and huge datasets [7,8,9]. At the same time, interest in depth cameras and, consequently, depth maps, has steadily grown in the computer vision community. Their increasing popularity has been supported by the spread of inexpensive, but still accurate, active depth sensors and their ability to operate in dark or in low-light conditions, thanks to the presence of infrared light or laser emitter [10]. For instance, in the automotive scenario [11,12], depth sensors represent an effective solution to run non-invasive and vision-based algorithms, such as face verification [13], head pose estimation [14], or gesture recognition [15]. More generally, starting from the first release of the *Microsoft Kinect* device, depth cameras have enabled new interaction modalities between the users and the environment. Gaming [16], smartphones [17], health care [18], and human-computer interaction [19] are just some other application fields where depth sensors have been used in addition or in replacement of the RGB cameras.

However, the different building technologies of depth sensors—e.g., *Structured Light* (SL) and *Time-of-Flight* (ToF) to cite the most common—hinder the efficacy of deep learning-based models when working with depth maps acquired from different depth sensors or even with the same technology, but in different acquisition setups. Indeed, the problem of cross-dataset and cross-device generalization is very critical with depth data, especially with deep learning approaches.

Generally, the problem is mitigated in the RGB domain, in which intensity images, from the visual point of view, are similar across sensors and huge datasets that are composed of images acquired by different cameras are available.

**Figure 1:**
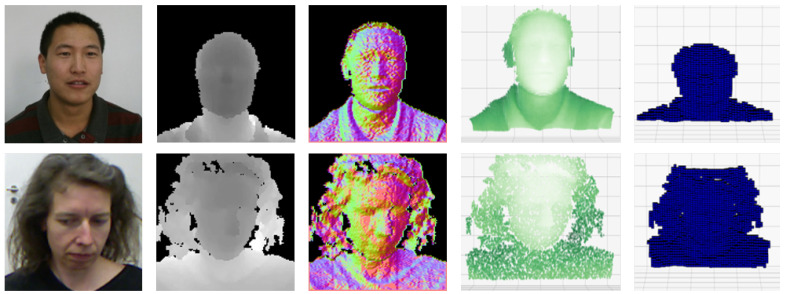
Sample images of different depth representation taken from *Lock3DFace* dataset [21] (*Time-of-Flight*, first row) and *Biwi* database [22] (*Structured Light*, second row). From the left, the RGB, depth and normal images, point clouds, and voxels are reported.

**Figure 2:**
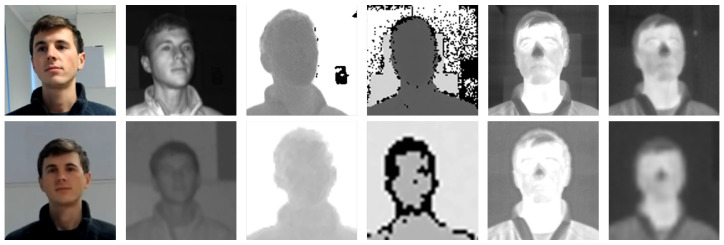
Images from the *MultiSFace* dataset. In the first row, sensors are placed near the subject (1 m), while in the second row sensors are placed far (2.5 m). Starting from the left: RGB and infrared images, high- and low-resolution depth maps, and high- and low-resolution thermal images. Section 3.3 reports further details. From the comparison, it is possible to evaluate how much the distance from the acquisition device and sensor resolution influences the collected data.

More specifically, the use of depth maps in combination with deep learning methods presents the following issues:

- The difference between depth maps acquired with different devices is significant, in terms of visual appearance (holes, shadows, noise), accuracy and detail preservation [20] (as it can be seen in Figure 1 and Figure 2).
- The same device is subject to environmental conditions, although the depth map should be independent of them; for example, it collects different data when facing direct sunlight or when the distance of the target from the device varies significantly. In the latter case, changes on the target distance affect not only the scale factor, but also the pixel values itself, the depth map quality, and the level of noise.
- Mixed datasets, i.e., the dataset acquired with different types of depth devices, still not publicly available. Moreover, the majority of the existing datasets are collected in a very limited number of acquisition settings, for instance using a single depth sensor for all of the the collected sequences. Thus, the generalization capabilities with respect to different devices and scenarios are often not analyzed in the literature.

Indeed, most of the available methods in the literature are task-tailored on a specific sensor, only performing intra-dataset tests, i.e., training and testing the proposed algorithms on the same data collection. Moreover, they usually use deep learning approaches to analyze depth maps that are represented as gray-level images, ignoring the intrinsic three-dimensional (3D) information that is embedded in depth data.

In this paper, we study the use of depth maps and deep neural models for the face recognition task, in search of the depth map representation that maximizes the recognition accuracy and better generalizes on unseen data. In particular, we compare different representations of depth data (depth images, normal images, point clouds, and voxels, as shown in Figure 1), pre-processing techniques (normalization, equalization, filtering, and hole filling), sensor technology (SL and ToF), and face-to-camera distance, in a comprehensive analysis.

The proposed comparison mainly focuses on the output of the active depth devices, which have a limited and well-defined maximum range; other types of sensors, such as stereo cameras, 3D scanners, and LiDARs are out of the scope of this paper.

Summarizing, the main contributions of this work are the following:

- We provide the first rigorous extensive analysis of depth data representations for the face recognition task, testing the performance and generalization capabilities on four depth-based public datasets.
- We investigate the use of data pre-processing, such as filtering, equalization, and hole filling, and normalization on depth images, often exploited in the depth-based literature methods.
- We evaluate different sensor technologies, SL and ToF, and the impact of subject distance and device resolution by proposing a new dataset, called *MultiSFace*, which includes more than 11k frames that were captured with two different synchronized depth sensors at different distances.

The experimental results suggest that normal images and point clouds that are computed from depth maps, even though rarely used in literature, are the best choice for achieving the highest accuracy and generalization in the face recognition task.

To the best of our knowledge, there are no existing works analyzing the use of different depth map representations and neural architectures for the face recognition task in the intra- and cross-dataset setting. Similar works [23] only address different representations of synthetic full 3D models of objects, in particular for object recognition and 6DoF estimation.

## 2. Different Representations of Depth Maps

Depth sensors provide data in several formats, which can be represented as depth maps. Formally, a depth map can be defined as $D_{M}=\langle D,K\rangle$, where $D=\{d_{ij}\}$, with $d_{ij}\in \left[\;0,R\;\right]$, is a matrix of distance values between 0 and the maximum measurable range *R*, and K is the perspective projection matrix that is obtained with the intrinsic parameters of the sensor. More specifically, $d_{ij}$ is the distance between the optical center and plane parallel to the image plane containing the physical point. The 3D coordinates of each captured point can be recovered from D and K, and then used to compute point clouds and voxels. Most of the computer vision algorithms do not directly exploit $D_{M}$ as input, but they convert $D_{M}$ in depth images, voxels, or point clouds, as described in the following paragraphs.

### 2.1. Depth Maps as Depth Images and Normal Images

The depth image is the most used representation of range data and it is a mere re-quantization of the D distance matrix. A depth image $I_{D}$ is encoded as a one-channel gray-scale image, in which the intensity of each pixel represents the quantized version of $d_{ij}$. This representation is usually referred as depth image, as well as range or 2.5D image. Spatial resolution, depth precision, and data format strictly depend on the acquisition device. Frequently, 8-bit gray-scale image formats are used to increase compatibility and facilitate viewing. Consequently, the computed depth image looses the full 3D content of the original depth map, in exchange for a 2D representation, which is easier to manage.

Thus, several works combine the use of Convolutional Neural Networks (CNNs) and depth images as standard intensity images addressing a variety of tasks. In [14,24], depth images and CNNs are proposed to regress the 3D head pose. In [14], depth images are also used to compute optical flow and to generate gray-level facial images by GANs. In [25], several pre-processing steps are applied on depth images, including hole filling (to reduce the areas with invalid depth values) depth range normalization (based on the nose tip detection), and outlier removal. Hu et al. [26] present a method for boosting depth-based face recognition through the combined use of high-quality depth data that were acquired by a 3D scanner and depth images. In [27], a Siamese network that processes pairs of facial depth images is proposed without exploiting any specific image pre-processing algorithms. Depth data are simply normalized to have zero mean and unit variance. Some pre-processing methods for depth images are proposed in [28], including nose tip detection for face crop and head pose correction. Sometimes, depth images are used in combination with other types of data that were obtained from depth or RGB-D devices, like intensity images [29,30] or human body joints [16,31].

However, the visual appearance of depth images is not device-invariant and it is strictly related to the sensor technology and the acquisition setup. Moreover, pre-processing steps, which are useful on intensity images, could partially or completely remove the metric depth information and destroy the 3D consistency.

We define a normal image as a matrix of pixels with three channels ${\hat{I}}_{N}=\left\{{\hat{v}}_{ij}=\langle {\hat{v}}_{x},{\hat{v}}_{y},{\hat{v}}_{z}\rangle \right\}$, where each pixel encodes the (x,y,z) components of the estimated surface normal vector in that point.

In our work, we follow [32] in order to obtain an estimation of surface normals starting from depth images. Specifically, given the depth matrix D, it is possible to indicate with Z(x,y) its pixel values. Subsequently, the direction $d=\langle d_{x},d_{y},d_{z}\rangle$ of normals is computed as:

(1) $$d=\left(-\frac{\partial Z(x,y)}{\partial x},\;-\frac{\partial Z(x,y)}{\partial y},\;1\right)$$

where ∂Z(x,y)/∂x,∂Z(x,y)/∂y are the gradients obtained on the depth in the *x* and *y* directions [33]. These directions can be calculated as:

(2) $$\begin{array}{c} \frac{\partial Z(x,y)}{\partial x}\approx Z\left(x+1,y\right)-Z\left(x,y\right)\\ \frac{\partial Z(x,y)}{\partial y}\approx Z\left(x,y+1\right)-Z\left(x,y\right) \end{array}$$

Finally, the vector $\hat{v}$ is the result of the following normalization [34]:

(3) $$\hat{v}=\frac{1}{B}\left(d_{x},d_{y},1\right),\;\;B=\sqrt{d_{x}^{2}+d_{y}^{2}+1}$$

It is worth noting that only few works exploit normal images directly obtained from depth maps. The discriminative content of normal images [35,36] can be exploited, even in combination with the depth images for the face recognition task [25].

### 2.2. Depth Maps as Point Clouds

Depth maps can be converted into the corresponding 3D point cloud with coordinates that are defined on the camera reference frame. Formally, a point cloud can be represented as an unordered set of points $P=\left\{p_{k}=\langle p_{k_{x}},p_{k_{y}},p_{k_{z}}\rangle \right\}$, where a generic point $p_{k}$ is a vector containing its 3D coordinates [37]. The conversion from the depth map to the point cloud can be defined as

(4a)pkx=(xi−cx)·Z(xi,yj)fx(4b)pky=(yj−cy)·Z(xi,yj)fy(4c)pkz=Z(xi,yj)

where the 3D point $p_{k}=\langle p_{k_{x}},p_{k_{y}},p_{k_{z}}\rangle$ corresponds to the value that is sampled over the depth map at a generic location $\left(x_{i},y_{j}\right)$ and the constants $f_{x},f_{y},c_{x},c_{y}$ are the elements that define the camera intrinsic parameters K (assuming that the pixels of the sensors are squared, i.e., having skew s=0). In practice, many of the depth sensors (e.g., Microsoft Kinect, Pico Zense) can also directly provide the 3D point cloud in addition to the depth maps as an option.

Because point clouds are unordered, with a variable length *n*, and sparse in the 3D space, they are more difficult to be exploited as input for deep networks. Moreover, because depth maps only contain 2.5D information, the extracted point cloud contains partial 3D information, i.e., a single view of the 3D scene. Consequently, to the best of our knowledge, no works propose using point clouds directly obtained from depth maps for the face recognition task.

On the other hand, point clouds are adopted for the 3D object recognition task, often on synthetic datasets, as in the work of Qi et al. [37]. The proposed network *PointNet* is directly fed with unordered 3D point sets and it is robust to input rotation, corruption and perturbation. Its evolution, called *PointNet*${}^{++}$ [38], consists in a recursive use of the *PointNet* model on subsets of neighboring points and it is able to learn local features with increasing contextual scale. Similar to [38], later works propose increasing the model capacity stacking hierarchically *PointNet* [39,40]. Other recent works [41,42] propose the use of local convolutions on point clouds. Still, the accuracy improvement with respect to earlier work is limited. It is worth noting that deep learning-based models that deal with point clouds are often computationally inefficient [43,44] and they require a great amount of memory. Only recently, [43] investigated how to reduce the memory consumption and inference time.

### 2.3. Depth Maps as Voxels

A voxel is a point-wise three-dimensional volumetric representation, the 3D equivalent of a 2D pixel in standard intensity images [45].

In the literature, the term voxel is also used to represent a 3D volume that is defined as tridimensional matrix $V^{m}=\left\{v_{ijh},\;\;i,j,h=1,...,m\right\}$, where *m* is the number of elements for each side of the 3D cube and each element $v_{ijh}\in \left\{0,1\right\}$ is a binary value, with 0 representing an empty space and 1 an occupied one. In details, a 3D point cloud *P* can be converted in a voxel $V^{m}$ with the following procedure. Defining a 3D cube with side length *L* centered in $p_{c}=\left(p_{c_{x}},p_{c_{y}},p_{c_{z}}\right)$ (which usually corresponds to the center of the point cloud) and the number *m* of binary voxels for each side of the cube, the 3D volume is split into m×m×m binary elements of side $l=\frac{L}{m}$. Each binary element $v_{ijh}$ represents the presence of at least one point lying inside its corresponding 3D volume $s_{ijh}$ of side *l*:

(5) $$v_{ijh}=\left\{\begin{array}{cc} 1 & \exists \;p_{k}\in P\;|\;p_{k}\in s_{ijh}\\ 0 & \mathrm{otherwise} \end{array}\right.$$

In other words, $v_{ijh}\in V^{m}$ is a binary value that indicates whether at least one point of the point cloud lies in the 3D volume $s_{ijh}$ corresponding to its cell. Unlike voxels computed from 3D models, which report the whole volume of the 3D object as occupied, only the voxels that correspond to the external visible surface of the object are identified from the depth maps, i.e., only the 3D data that the depth sensor is able to acquire.

At time of writing, only a few works propose analyzing voxels obtained from depth maps with deep approaches. Moon et al. [46] propose the use of a specific 3D CNN, called *V2V-PoseNet*, to tackle hand and human pose estimation. A voxel-to-voxel architecture is developed to predict 3D heatmaps, from which 3D coordinates of hand keypoints or human body joints are obtained.

A considerable number of methods are based on voxels obtained from 3D scanners or LiDARs. For instance, in [47] voxels are the input of a supervised 3D CNN for the object detection task. The experimental results are collected processing voxels that were obtained from 3D scanners. Zhou and Tuzel [48] propose *VoxelNet*, a generic detection network able to work with voxels obtained from LiDAR data. Recently, Riegler et al. [49] propose partitioning sparse 3D data through a set of unbalanced octrees, in which each leaf node stores a pooled feature representation. They test the proposed method on the 3D object classification and orientation estimation tasks.

In general, the use of voxels, together with deep learning models, is limited, since a reference point, i.e., the point around which the 3D space (usually a 3D cube) is sampled, is needed. Furthermore, it is necessary to define the volume of 3D space around the reference point and the size of the single voxels, i.e., the level of quantization. All of these elements deeply influence the final performance of systems based on voxels used as input data [46,50].

## 3. Methodology

In this paper, we analyze the use of depth maps for deep face recognition. We aim at identifying the combination of data representation, a pre-processing/normalization technique, and deep learning model that obtains the highest recognition accuracy in both the intra- and the cross-dataset setting. In this section, we characterize this analysis, from the problem statement and the deep learning models to the datasets and pre-processing techniques.

### 3.1. Problem Statement and Experimental Setting

We address the face recognition task as a face identification problem, where a single depth map of an unknown person, i.e., the probe, is compared to a gallery of known candidates in a closed-set scenario. In this setting, the recognition model compares the probe with each gallery identity, i.e., a one-to-many comparison, and then outputs a single label that represents the predicted identity of the probe. Given the predicted identity, we compare different approaches in terms of recognition accuracy (i.e., top-1 recognition rate) and compare different deep architectures in terms of computational complexity.

Within the different experimental settings (i.e., the different combinations of data representation, pre-processing and normalization steps, and deep model), we employ the same training procedure. Each model is trained on the train split of the selected dataset for 50 epochs (that we empirically observe as a valid upper-bound limit), while using the Categorical Cross-Entropy (CCE) loss and Adam optimizer. After every epoch, the validation accuracy is evaluated and, if higher than any validation accuracy obtained so far, the model parameters are saved (and later used for testing).

In the testing phase, we discard the last classification layer and compare the probe and gallery depth maps computing the cosine similarity between the deep features that were extracted by the networks [51]. For every probe, we select the predicted identity as the gallery candidate corresponding to the maximum similarity.

### 3.2. Deep Learning Architectures

Well-known and representative deep learning-based models are selected for the evaluation part. For depth maps used as single-channel images, we exploit the models *VGG-16* [6], *ResNet-18* [4], and *Inception-v3* [5]. Voxels are used in combination with *VoxNet* [47], *R3D* and *R(2+1)D* [52], while *PointNet* [37] and *PointNet${}^{++}$* [38] are employed for point clouds.

Deep Networks are implemented in *PyTorch* and adapted for the specific task of face recognition from depth data, in terms of input channels and final classification layer. For instance, the first layer of networks used to analyze the depth images is adapted to support a single-channel input, while the classification part of PointNet and PointNet${}^{++}$ is used and the segmentation branch is discarded. For a fairer comparison between models (image-based networks are often pre-trained on bigger datasets), all of the networks are trained from scratch.

In all of the experiments on every dataset, we employ the same input format, as detailed in the following. Regarding the 2D CNNs, the input images are resized to the resolution of 128×128 pixels and the background behind the human face is filtered out, if present. The images are represented with single-channel images while using the 16-bit format. The depth values are expressed in mm. When considering the point clouds, we compute them from the depth maps, as detailed in Section 2.2. We consider, as valid, all of the points with a non-null depth value and feed them to the point cloud-based networks. The maximum number of points is set to 16,384. When using the 3D voxels, we obtain them from the point clouds of the human face. We centered the 3D volume at the point cloud center (computed as the mean of the coordinates of all the points) and set a cubic side L=400 mm. The number of voxels per side *m* can be 32 or 64, as defined in the experimental results.

### 3.3. Datasets

Although the spread of depth sensors is still limited with respect to RGB ones, depth-based datasets containing faces are already available in the literature. Each of them has been acquired using a single depth sensor, e.g., *Structured Light* (SL) or *Time of Flight* (ToF).

Among them, we have selected two datasets that were acquired with the first version of the *Microsoft Kinect* sensor, based on the SL technology, and two datasets that were acquired with the second version of the same device, based on the ToF technology. We preferred to exclude other available datasets that contain a limited number of subjects (e.g., [53]), frames (e.g., [54]), unreliable depth data (e.g., [55]), or 3D facial models instead of depth maps (e.g., [56]).

Table 1 reports an overview of the chosen datasets, which presents, for each dataset, the sensor technology; the number of subjects, frames, cameras, and sessions; the level of complexity when considering the face recognition task (expressed as chance level); and, the number of different acquisition settings. We split the data inro train, validation, and test sets using, whenever possible, different sessions/sequences for each subset. We aim at obtaining a fair subdivision, i.e., the use of different sessions/sequences for each subset while including samples of each person in the training set. When the official splits conform to this policy, we used the official train, validation, and test subsets. We also note that each employed dataset was acquired with a different procedure and thus requires a subdivision that is based on its structure, yielding a different number of recordings in different settings for each dataset. Table 2 reports the number of frames belonging to each split.

In the following, we present the selected datasets in detail:

- *Biwi*: introduced in [22], it contains approximately 15 k depth frames of the upper body part of 20 subjects, acquired with the first version of the *Microsoft Kinect* (SL). Each subject records a sequence during which they were asked to rotate the head spanning all of the head angles they were capable of. In our experiments, we use the first half of each sequence as training set. The second half is randomly shuffled and split in the validation (40%) and the test set (60%). This is mandatory, since there is only one session per most of the subjects and each session contains a scripted set of head movements. Four subjects are recorded twice. We do not use the additional recordings of these subjects.
- *CurtinFaces*: released in [28], it addresses the task of face recognition under varying expressions, poses, illumination sources, and disguises. It uses the first version of the *Microsoft Kinect* (SL) and it consists of 5044 images that were recorded from 52 subjects (97 images per subject). In our experiments, we use 18 images per subject as training set (as in the original paper), 8 images per subject as validation set, and the remaining images as test set (i.e., 71 images per subject). We refer the reader to [28] for more details regarding the training split. The validation split is sampled, including a different pose for every different expression and two illumination variations, in order to cover the dataset distribution.
- *Pandora*: as presented in [14], this dataset was collected for the head pose estimation task, but it has also been exploited for the face verification task [13,27]. Acquired using the second version of the *Microsoft Kinect* (ToF), it contains 22 subjects and five sequences for each subject. The faces can be occluded by the presence of garments and extreme head poses. In our experiments, we use the sequences without garments and artificial occlusions (i.e., the first three sequences of each subject). In particular, for each subject, we use the first sequence as training set, the second one as validation set, and the third one as test set.
- *Lock3DFace*: published in [21], it consists of more than 300 k frames of 509 different subjects recorded with the second version of the *Microsoft Kinect* (ToF) in multiple acquisition sessions. It contains variations in poses, facial expressions, and occlusions, and each variation is performed multiple times (from two to six recordings). Moreover, 169 subjects have been recorded in separate sessions with a temporal step of up to 7 months. The dataset has been split in a training set, which is composed of the first recording of each type for each subject, a validation set, composed of the first frame of the other recordings, and a test set, composed of the remaining frames. We select the first recording of each type for each subject as a training set, regardless of the temporal session. Subsequently, since the number of recordings per variation is subject dependent (and vary from 2 to 6), we select the first frame of the additional recordings as a validation set and the following frames of each recording as test set.

In addition, we collected a new cross-device dataset for the evaluation of multi-device and multi-distance face recognition based on depth maps:

- *MultiSFace*: 31 subjects are acquired in three different poses—frontal, side, and back—at two different distances—near (1 m) and far (2.5 m)—through different depth devices at the same time, as shown in Figure 2. The first device, the *Pico Zense DCAM710* (https://www.picozense.com/), is a high-resolution depth camera that is based on the ToF technology that acquires depth frames with a resolution of 640×480 pixels at 30 fps in a range of 0.2–5 m. The second sensor is a low-resolution depth camera, the *CamBoard Pico Flexx* (https://pmdtec.com/picofamily/), a ToF device more focused on portability, in terms of both lightweight (8g) and form factor (68×17×7.35 mm), than depth quality: as shown in the fourth column of Figure 2, a high level of noise and a limited resolution (171×224) are present in the operating range (0.1–4 m).
  The dataset also contains images that were acquired with additional devices: a high-resolution thermal camera (*Flir Boson 640* (https://prod.flir.it/products/boson/)), a low-resolution radiometric thermal camera (*Flir PureThermal 2* (https://groupgets.com/manufacturers/flir/products/lepton-2-0)), two RGB cameras with different resolution and image quality. Only depth frames from the sequences containing frontal views are used in this work.

The *MultiSFace* dataset allows for investigating the impact of using different depth sensors at varying distances on the face recognition accuracy. To the best of our knowledge, *MultiSFace* is the first publicly available dataset, in which each subject is acquired with different synchronized depth (and thermal) sensors. MultiSFace is designed as a testing dataset, in order to make an extremely challenging benchmark on multi-modal face recognition available to the research community. Specifically, we conceive this dataset with the goal of providing a tool to investigate the cross-device and cross-distance issues. The *MultiSFace* dataset is available at https://aimagelab.ing.unimore.it/go/multisface.

### 3.4. Pre-Processing Techniques

We select common image pre-processing techniques that are applied on depth images in the literature [21,57,58], such as filtering, histogram equalization, and hole filling. We individually apply them on $I_{D}$.

Filters areoften applied to reduce the high level of noise caused, for instance, by external light sources and the use of an infrared emitter [20]. To this aim, in the tests we included a linear filter (Gaussian), a non linear filter (Median), and a data-dependent, thus not shift-invariant, filter (Bilateral).

Histogram equalization is applied to enhance the contrast in the intensity images and it can be used to stretch very similar values in depth facial images. Specifically, we consider the standard equalization and the *Contrast-Limited Adaptive Histogram Equalization* (CLAHE) [59] algorithms.

Depth maps often present pixels with missing or spurious depth values, due to specular or low albedo surfaces: typical parts with invalid values are hair and eye areas. Additionally, shadows, which are created by the disparity between the sensors and infrared emitter, contain missing values. Therefore, some works propose using hole filling (in-painting) techniques, replacing invalid data. In our work, we adopt the hole filling procedure that is described in [60].

**Figure 3:**
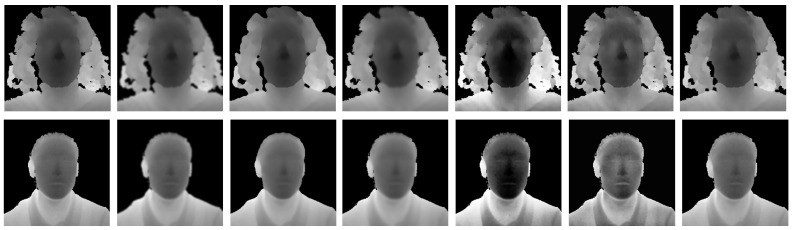
The sample images taken from *Biwi* [22] (first row) and *Lock3d* [21] (last row) showing the visual results of the pre-processing steps. On the left, the original depth image is reported. Then, we show the results of the following operations: gaussian blur, median blur, bilateral filtering, histogram equalization, *Contrast-Limited Adaptive Histogram Equalization* (CLAHE), and hole filling. The images are converted to 8-bit format for visualization.

We report some visual results of these pre-processing techniques in Figure 3.

### 3.5. Data Normalization

Generally, data normalization is a key element during the training process of deep learning models with intensity images [61]. In our case, we test the following normalization procedures on depth data:

(6) $$f_{1}\left(x\right)=x-\mu_{x}$$

(7) $$f_{2}\left(x\right)=\frac{x-\mu_{x}}{\sigma_{x}}$$

(8) $$f_{3}\left(x\right)=\frac{x-min(x)}{max(x)-min(x)}$$

where $\mu_{x}$ and $\sigma_{x}$ are the mean and standard deviation operations. When applied to depth images, *x* is the set of valid pixel values (i.e., pixels that are not null, due to an invalid depth estimation or that do not exceed the maximum depth range of the device). Point clouds are normalized by applying the operation on each axis, independently. Equation (6) zero-centers the data/point coordinates, Equation (7) gives data/point coordinates with zero mean and unit variance, while Equation (8) outputs the values in the range [0,1].

## 4. Intra-Dataset Experiments

Intra-dataset experiments are carried out on individual datasets, each split into training, validation, and testing sets. Thus, models are trained and tested with data that were acquired by the same depth device and environment, then similar from a visual and quality point of view. These experiments are focused on the investigation regarding the use of depth data and deep architectures, in terms of accuracy in face recognition, not considering generalization capabilities on different datasets and depth technologies. We report the results in terms of recognition accuracy, as described in the beginning of Section 3, while using depth and normal images, voxels, and point clouds in Table 3, Table 4 and Table 5.

We report the best performing pre-processing and normalization steps, which are individually applied, as described in Section 3.4. Specifically, for the depth images, we include the Gaussian filter (**F**) for filtering, Equation (8) for data normalization, and the histogram equalization (**E**), while **H** denotes the hole filling procedure. $I_{N}$ represents the use of normal images as input data. For the point cloud, the data normalization referred as $P_{N}$ is computed, as in Equation (6). For the voxels, two different sizes (m=32 or m=64) are evaluated.

Looking at the results of image-based methods (Table 3), in general filtering, the equalization and hole filling procedures do not introduce clear benefits, even if they are often exploited in literature, as highlighted in Section 2. Therefore, the additional computational load that is introduced by them is not justified by a corresponding increase of accuracy. Instead, data normalization generally maintains or improves the results, in particular on ToF data.

Nevertheless, the results show that normal images are the best data representation for recognizing faces while using CNNs in most cases. When compared to depth images, normal images do not contain the absolute distances of the target points, but they explicitly express 3D information that is related to the 3D shape of the captured scene. Thus, we hypothesize that the resulting representation is more suitable for the face recognition task while using depth devices.

Deep architectures based on point clouds and voxels generally achieve worse results than image-based approaches, as it can be seen in Table 4 and Table 5. In the case of point clouds, the results show that data normalization is a key element to achieve a good level of accuracy (especially with *PointNet*${}^{++}$), while experiments with voxels show that the attained accuracy is not dependent on the network architecture and voxel size. Even from a computational point of view, CNNs are usually the best choice in terms of memory usage and inference time.

## 5. Cross-Dataset Experiments

Cross-dataset experiments are carried out considering two datasets at a time, one for training the deep models and one for testing. Probe and gallery data are both extracted from the second dataset. Each experiment is referred in the form “$D_{1}\to D_{2}$”, which means that the model is trained on the dataset $D_{1}$ and tested on $D_{2}$. Compared to the intra-dataset case, these tests are focused on the generalization capabilities of deep models, in particular when the two datasets have been acquired while using different sensor technologies or in different acquisition settings.

In Table 6, we report the most interesting results of the cross-dataset evaluation, obtained with *ResNet*, *R3D*, and *PointNet*${}^{++}$ for depth images, normal images, voxels, and point clouds. As in the intra-dataset setting, the results are expressed in terms of recognition accuracy, following what reported at the beginning of Section 3. The left part of the table contains results that were obtained using train and test datasets that were acquired with the same sensor technology, while the right part contains experiments in which the sensor technology of the test dataset is different from the one of the training dataset. For the sake of comparison, the best results that were obtained in the corresponding intra-dataset experiment are reported as "best (intra)". The reference values included in the table are the ones obtained using $D_{2}$ for both the training and testing and collected from Section 4.

First of all, we note that point cloud-based methods are the best choice in the cross-dataset setting, even if point clouds that are computed from depth maps are rarely used in the literature for the face recognition task. They achieve the best accuracy with both same and different sensor technologies, as confirmed by both the absolute accuracy and the minor performance drop when compared with the intra-dataset references, as shown in Table 6. This finding confirms that this data representation is more independent from the acquisition sensor and that the point cloud-based models are less prone to overfit on the training dataset. Therefore, point clouds should be used when the testing data are acquired by different or unknown depth sensors. We believe that the performance discrepancy between the intra-dataset setting and cross-dataset one reveals a potential difficulty in assessing the quality of point cloud-based methods. In fact, most of the experiments that are reported in the literature do not deal with cross-dataset tests and may only observe unsatisfactory results in the intra-dataset setting.

Regarding the other depth map representations, normal images analyzed with CNNs obtain higher accuracy when compared to depth images and voxels, thus confirming that surface normals are an informative and invariant representation of depth maps for the face recognition task.

As it can be noted, the architectures trained on *Pandora* achieve better results than the ones trained on *Lock3DFace* whether tested on *Biwi* or *CurtinFaces*, in particular when considering normal images and point clouds. Because the main differences between Pandora and Lock3D are the number of frames with different poses (higher in the former) and the number of subjects (higher in the latter), we hypothesize that, for the face recognition on 3D representations of depth data, the head pose variability of the training set is more crucial than the number of different identities.

## 6. Cross-Device and Cross-Distance Experiments

The proposed dataset *MultiSFace* contains data that were acquired from diversified positions by two different depth sensors. Therefore, it could be used to run an additional set of challenging experiments. In fact, it can be employed to evaluate the recognition accuracy when the gallery set and the probe data are collected by different devices or at different sensor-subject distances.

We run this set of experiments employing architectures that were trained on the *Lock3DFace* dataset (we used ResNet for $I_{D}$, PointNet${}^{++}$ for $P_{N}$, and R3D for $V^{32}$). We evaluate the recognition accuracy using two ToF sensors (having different resolutions), labelled as High Resolution (HR) and Low Resolution (LR), and two different sensor-subject distances, labelled as Near (N) and Far (F). It should be recalled that, since depth maps are acquired by depth devices, the sensor-subject distance directly affects their quality, in terms of noise and point density. Therefore, even if some data representations are distance-invariant (e.g., depth normals, voxels, and point clouds), the depth data acquired by the sensors are not.

Table 7 reports the results in terms of recognition accuracy. The better generalization capabilities of the point cloud representation and PointNet${}^{++}$ are highlighted. However, the tested approaches do not reach satisfactory recognition accuracy in these challenging cases. Image-based methods achieve results around 4–6%, which are only slightly higher than the chance level, while the voxel representation can be suitable for the cross-device scenarios, since the voxel quantization filters out the differences in the resolution and quality between the sensors. This holds at the Near (N) distance, where both of the sensors acquire sufficiently-precise depth maps, while it does not hold at the Far (F) distance, due to the noisy sparse data acquired by the sensors, especially the low-resolution device.

## 7. Discussion

In this section, we summarize the main considerations that follow from the intra- and cross-dataset experiments and from the additional analysis obtained on the *MultiSFace* dataset.

First of all, we observe that, in general, approaches that rely on depth images and CNNs are limited in terms of the generalization capabilities. That is, a substantial performance drop occurs when these models are tested with depth data that differ from the training one (as data acquired by the same depth sensor in a different setting or another sensor with the same or a different building technology). On the other hand, normal images represent the best choice in order for obtaining higher accuracy in a cross-dataset scenario while using CNNs. However, they are employed in a minor part of literature work.

Moreover, the results clearly show that point cloud-based representations and architectures are the best option in terms of generalization capabilities when the training and testing data do not belong to the same dataset (i.e., the data are collected in different acquisition setups). Because similar experiments are not available in the literature, the reported results can be considered a baselines for future investigation in this research field.

When considering the intra-dataset setting, the results show that the face recognition task can be carried out while using depth maps, even if they only contain geometrical information (in contrast to intensity images that contain shapes, colors, and textures). However, the generalization capabilities of these architectures have still not been tested on more challenging settings, i.e., when the probes and the gallery set are acquired with different depth devices or in different scenarios. These types of experiment can not be carried out using existing datasets since intra-dataset experiments contain data that are captured by a single depth sensor, while cross-dataset experiments are not possible (because different subjects are included in every dataset).

To this end, we have collected the proposed *MultiSFace* dataset and, in Section 6, we have reported the results obtained on it using probes and gallery sets acquired by different depth sensors and at different sensor-subject distances. These results confirm that 2D representations of depth maps, which are processed with CNNs, are not a suitable solution for cross-device and cross-distance settings. They also show, in line with previous findings, that point cloud-based representation and architecture are the optimal solution in the majority of the tested settings.

However, we want to highlight that the accuracy on the *MultiSFace* dataset is, without any doubt, quite low, showing the challenging nature of the recognition task in these scenarios, which was made possible by this particular dataset. In contrast to the high recognition accuracy obtained in the single-sensor single-dataset scenario, the face recognition task carried out in the wild using several depth sensors in different acquisition settings is far from being solved. We believe that this dataset can inspire and be an interesting benchmark for future investigations regarding face recognition with depth maps that are focused on generalization capabilities over depth sensors and data.

## 8. Computational Complexity

The recognition accuracy is not the only element to be taken into account during the development of real-world face recognition systems. Therefore, in this section, we report an analysis of the computational complexity of the investigated approaches. In particular, we report the number of parameters, the memory consumption, and the inference speed of each method shown in Table 8. All of the deep models have been implemented while using the PyTorch framework [62] and then tested on a computer equipped with an *Intel(R) Core(TM) i7-7700K* and a *NVidia GTX 1080Ti*.

The first three rows of Table 8 involve CNNs relying on 2D input images, and then voxel-based approaches are reported in the central rows and the last two rows contains the point cloud-based models. As expected, the number of parameters of 2D CNNs is correlated with the memory occupation: in this context, the VGG-16 model has the highest number of parameters and, then, the highest RAM occupation. Nevertheless, its inference time is remarkably low, which is probably thanks to the level of optimization for the convolutional operations in the PyTorch framework [63]. The same analysis also holds for voxel-based methods. When considering PointNet and PointNet${}^{++}$, the former requires a little amount of memory and a sufficiently low inference time while the latter represents an exception having a very high inference time. We believe that this is caused by the several clustering operations, still not optimized on GPUs, needed by the architecture.

From a general point of view, we observe that a depth-based face verification system that is implemented with one of the analyzed architectures can have real time performance on a workstation and that the RAM usage is low when compared to the typical memory size of commercial GPUs (6–12 GB).

## 9. Conclusions

In this paper, an extensive comparison on the use of depth maps and deep learning-based approaches is conducted. We investigate how data representations, network architectures, pre-processing, and normalization techniques affect the accuracy in the face recognition task using depth maps. We present the results that were obtained on four public datasets with multiple intra- and cross-dataset tests that suggest that depth maps should not be represented and treated as standard images. The results show that pre-processing and data normalization techniques, applied in combination with convolutional networks, reduce the 3D content of the depth data, making the corresponding systems less capable of generalizing and transfering to other depth domains (e.g., different sensors and acquisition setups). Representations that are based on normal images and, in particular, point clouds alleviate this problem and result in models with better generalization capabilities. We also present a new challenging dataset, called MultiSFace, which contains facial data that were acquired by different synchronized sensors and in different conditions (i.e., at different sensor-subject distances). The results obtained on this dataset reveal the need for a proper face recognition method that is invariant to the acquisition sensor and setting and, in general, capable of fully exploiting the 3D content of depth maps. 

## Figures and Tables

**Table 1:** Datasets that were selected for the proposed analysis. DT is the depth technology, #subjs is the number of subjects, #frames is the number of depth frames and #cams the number of depth camera used. The chance (level) is the accuracy with random predictions. Settings correspond to the position of the subject w.r.t. the acquisition device. Sessions is the number of different acquisitions per subject.

Name	Year	DT	#Subjs	#Frames	Chance (%)	#Cams	Settings	Sessions
Biwi [22]	2011	SL	20	15k	5.0	1	1 (near)	1 or 2
CurtinFaces [28]	2013	SL	52	5k	2.9	1	1 (near)	17
Lock3DFace [21]	2016	ToF	509	300k	0.2	1	1 (near)	8 to 16
Pandora [14]	2017	ToF	22	125k	4.5	1	1 (near)	5
MultiSFace	2020	ToF	31	11k	3.2	2	2 (near, far)	2

**Table 2:** Training, validation, and testing splits adopted for each dataset. Frames are split following the procedures described in Section 3.3.

Name	Total Depth Frames	Training	Validation	Testing
Biwi [22]	15 k	6.6 k	2.6 k	3.9 k
CurtinFaces [28]	5 k	0.9 k	0.4 k	3.7 k
Loc k3DFace [21]	300 k	12.2 k	2.7 k	17.8 k
Pandora [14]	125 k	9.3 k	7.4 k	9.5 k
MultiSFace	11 k	-	-	3.5 k

**Table 3:** Intra-dataset results, in terms of recognition accuracy, using depth and normal images ($I_{D}$ and $I_{N}$). Pre-processing steps applied on depth images, i.e., filtering ($F(I_{D})$), hole filling ($H(I_{D})$), equalization ($E(I_{D})$), and data normalization ($N(I_{D})$), are also reported for depth map representation.

	Biwi [22]						CurtinFaces [28]					
Model	$I_{D}$	$F(I_{D})$	$H(I_{D})$	$E(I_{D})$	$N(I_{D})$	$I_{N}$	$I_{D}$	$F(I_{D})$	$H(I_{D})$	$E(I_{D})$	$N(I_{D})$	$I_{N}$
VGG [6]	32.5	33.6	32.9	29.9	26.6	43.1	60.5	57.7	57.4	63.4	57.5	66.5
Inception [5]	60.9	56.8	52.9	45.4	50.8	66.8	29.5	40.0	34.0	33.7	38.6	42.2
ResNet [4]	61.5	64.4	58.3	64.0	66.7	80.0	43.0	45.6	40.0	48.8	50.9	45.2
	**Lock3DFace** [21]						**Pandora** [14]					
Model	$I_{D}$	$F(I_{D})$	$H(I_{D})$	$E(I_{D})$	$N(I_{D})$	$I_{N}$	$I_{D}$	$F(I_{D})$	$H(I_{D})$	$E(I_{D})$	$N(I_{D})$	$I_{N}$
VGG [6]	54.6	53.4	55.2	61.3	54.9	62.1	51.6	51.2	47.2	54.0	51.3	57.4
Inception [5]	72.5	71.6	72.1	70.3	72.3	81.0	40.0	40.1	35.5	63.9	59.6	72.4
ResNet [4]	51.7	52.8	50.9	56.3	59.0	76.6	40.3	42.7	42.6	67.1	65.4	70.3

**Table 4:** Intra-dataset results, in terms of recognition accuracy, using point clouds *P*. $P_{N}$ represents the normalized point cloud computed while using Equation (6), as detailed in Section 3.5.

	Biwi			CurtinF.			Lock3D			Pandora	
Model	*P*	$P_{N}$		*P*	$P_{N}$		*P*	$P_{N}$		*P*	$P_{N}$
PointNet [37]	**60.5**	53.2		50.7	70.7		55.1	63.9		23.9	25.2
PointNet${}^{++}$ [38]	40.4	42.2		45.4	51.7		51.4	61.8		21.1	35.8

**Table 5:** Intra-dataset results, in terms of recognition accuracy, using voxels *V*. 32 and 64 specify the size *m* of the three-dimensional (3D) volume (see Section 2).

	Biwi			CurtinF.			Lock3D			Pandora	
Model	$V^{32}$	$V^{64}$		$V^{32}$	$V^{64}$		$V^{32}$	$V^{64}$		$V^{32}$	$V^{64}$
VoxNet [47]	**53.0**	49.2		78.0	73.7		67.8	69.1		36.6	37.2
R3D [52]	64.4	63.3		69.5	71.4		71.0	70.1		30.0	31.9
R(2+1)D [52]	61.4	58.8		40.0	67.1		68.7	68.5		31.8	37.6

**Table 6:** Cross-dataset results, in terms of recognition accuracy. The data type used in input is reported ($I_{D}$: Depth Maps, *V*: Voxels, *P*: Point Clouds), together to each dataset (C: *Curtinfaces* [28], B: *Biwi* [22], L: *Lock3DFace* [21], P: *Pandora* [14]), and each technology of depth sensors (SL: *Structured Light*, ToF: *Time-of-Flight*). $D_{1}\to D_{2}$ means “trained on $D_{1}$ and tested on $D_{2}$”.

	Same Sensor Technology				Different Sensor Technology							
	SL → ToF		ToF → SL		SL → ToF				ToF → SL			
Model	**C→B**	**B→C**	**P→L**	**L→P**	**C→L**	**C→P**	**B→L**	**B→P**	**P→B**	**P→C**	**L→B**	**L→C**
best (intra)	80.0	66.5	81.0	72.4	72.5	67.1	72.5	67.1	66.7	63.4	66.7	63.4
$I_{D}$	34.4	18.2	31.3	25.6	32.8	26.8	30.5	24.9	28.4	14.1	33.1	18.7
$I_{N}$	34.6	35.3	45.6	35.6	25.4	23.2	45.3	32.6	48.2	34.2	37.0	33.0
best (intra)	60.5	70.7	63.9	35.8	63.9	35.8	63.9	35.8	60.5	70.7	60.5	70.7
*P*	36.4	36.9	40.7	30.0	30.2	12.3	37.4	26.3	43.5	35.6	37.4	34.7
$P_{N}$	36.1	39.8	56.2	39.6	58.1	39.1	54.0	34.0	37.5	46.5	35.2	43.3
**best (intra)**	64.4	78.0	71.0	37.6	71.0	37.6	71.0	37.6	64.4	78.0	64.4	78.0
$V^{32}$	22.6	20.1	33.5	30.4	41.3	23.0	36.9	21.3	18.3	15.6	27.1	33.7
$V^{64}$	21.7	21.8	38.0	28.2	40.4	23.7	35.8	22.3	22.7	21.4	21.1	33.7

**Table 7:** Results on MultiSFace, in terms of recognition accuracy. Tests are carried using different gallery and probe data. In the left part, cross-distance tests (**N**ear and **F**ar distance) are reported keeping the sensor fixed. In the right part, cross-device tests (**HR** and **LR**, i.e., high and low resolution) are reported keeping the distance fixed.

		Cross-Distance				Cross-Device	
		**N → F**	**F → N**			**HR → LR**	**LR → HR**
**HR**	$I_{N}$	6.6	4.9	**F**	$I_{N}$	3.4	5.2
	$P_{N}$	16.7	13.9		$P_{N}$	9.2	7.5
	$V^{32}$	9.0	7.5		$V^{32}$	3.1	5.4
**LR**	$I_{N}$	4.6	4.4	**N**	$I_{N}$	6.4	2.7
	$P_{N}$	8.6	7.2		$P_{N}$	3.1	6.0
	$V^{32}$	4.4	5.0		$V^{32}$	10.8	8.0

**Table 8:** A comparison of the computational complexity of different methods. We report the number of parameters, the amount of memory (RAM), and the inference time that isrequired by the models, implemented in PyTorch.

Model	Parameters (M)	RAM (GB)	Inference (ms)
VGG-16	117.5	2.63	1.4±0.2
ResNet-18	11.2	0.76	2.2±0.1
Inception-v3	21.8	0.91	8.2±0.3
VoxNet	0.92	0.58	0.5±0.1
R3D	33.1	1.11	2.1±0.2
R(2+1)D	31.3	1.09	3.3±0.2
PointNet	0.95	0.74	4.8±0.1
PointNet${}^{++}$	0.81	1.17	226.5±5.5

## Data Availability

The *MultiSFace* dataset is available at https://aimagelab.ing.unimore.it/go/multisface.

## Abbreviations

The following abbreviations are used in this manuscript:

SL	Structured Light
ToF	Time-of-Flight
6DoF	Six Degrees of Freedom
CNN	Convolutional Neural Network
GAN	Generative Adversarial Network
CLAHE	Contrast-Limited Adaptive Histogram Equalization
HR	High Resolution
LR	Low Resolution
M	Million
ms	milliseconds
GB	GigaByte
